# Implementation of Psychodynamic Multifamily Groups for Severe Mental Illness: A Recovery-Oriented Approach

**DOI:** 10.3389/fpsyt.2021.646925

**Published:** 2021-04-07

**Authors:** Antonio Maone, Barbara D'Avanzo, Federico Russo, Rita Maria Esposito, Bozena Lucyna Goldos, Alessandro Antonucci, Giuseppe Ducci, Andrea Narracci

**Affiliations:** ^1^Dipartimento di Salute Mentale, Azienda Sanitaria Locale Roma 1, Rome, Italy; ^2^Istituto di Ricerche Farmacologiche “Mario Negri” IRCCS, Milan, Italy; ^3^Dipartimento di Psicologia, Sapienza Università di Roma, Rome, Italy; ^4^IRCCS Fondazione Santa Lucia, Rome, Italy

**Keywords:** community psychiatric care, family intervention, implementation, severe mental illness, recovery approach, Psychodynamic Multifamily Groups

## Abstract

**Background:** Among Family-Based Services for the treatment of severe mental illnesses, multi-family models gained particular attention, given the potential usefulness of mutual feed-back, motivation and encouragement among families.

**Methods:** The Psychodynamic Multi-Family Group Model has been proposed since 1997 in some Community Mental Health Services in Rome. Since 2011 multifamily groups are held weekly in all the six Districts of the Department of Mental Health that serves a population of more than one million people, and data have been collected since 2015 in three Districts. A total of 794 individuals attended the meetings in the period 2015–2019.

**Results:** Eighty-six percent of those who started, attended more than one meeting. The mean of occurrences of participation among patients was 18.6, among mothers 25.6 and among fathers 21.6. The 794 participants belonged to 439 family units, among which 180 comprised only the patient, 76 only parent(s) or other close person(s), and 183 comprised parent(s) or close person(s) with the patient. Patients participating alone were older than those of families who participated as a whole. Families including the patient showed the longest duration of attendance and the highest prevalence of a diagnosis of schizophrenia in the index patient. Families who had been attending the multifamily groups since a long time maintained a high rate of attendance.

**Conclusions:** Multifamily groups represent a setting where patients can meet with other people and professionals in a free still structured way, and with not strictly therapeutic objectives. The high number of patients who attended alone suggests that such participation corresponds to a self-perceived need of open and free setting facilitating sharing of problems and solutions. The good tenure of the interventions, the high participation, and the feasibility in the long-term suggest that multifamily groups can be implemented in the mental health services of a large city, are sustainable over many years, and can represent a valuable resource for many patients and families.

## Introduction

Contemporary mental health systems are still challenged by the need to offer adequate answers to people with severe and persistent mental disorders (1). In this area, knowledge and skills have increased enormously, also with the development and assessment of several consolidated models of psychosocial interventions. Among these, Family-Based Services gained particular attention (2). The importance of involving the families lies, on the one hand, in the fact that many mental health users live or are in regular contact with their relatives, often charged with the role of main caregivers with the related material and emotional burden (3); on the other hand, characteristics of the family atmosphere and communication patterns are associated with clinical course and outcome of the patient's disorder (4).

Family interventions may be based on different approaches and adopt different techniques still sharing several characteristics and aims: offering information about the disorder, supporting treatment adherence, assuming a non-pathologizing stance, strengthening communication, avoiding blaming, favoring empathy and mutual respect, sustaining personal growth and self-determination in all family members (5).

The most standardized and manualized models were also most frequently investigated and evaluated for their efficacy: meta-analyses confirmed their effect on relapse and readmission rates, treatment adherence, functional and vocational status, perceived stress among patients, levels of burden and distress and family relationships (6, 7). Their implementation is therefore considered evidence-based and recommended in clinical guidelines for the treatment of psychotic disorders (8, 9) as well as in other areas, like eating disorders (10) and other conditions.

In the last decades, models including more than one family in the same session of treatment gained particular attention (11, 12). One of the potential strengths of such model, compared to other individual or single family approaches, may reside in the mutual feed-back among families being more effective in enhancing support, motivation and encouragement than the therapists' action (13). Amongst such models, the Psychodynamic Multi-Family Group Model originates from the thought and the experiences of Jorge Garcia Badaracco (14), Argentinian psychiatrist and psychoanalyst, who worked in the psychiatric hospital of Buenos Aires since the 1960s. He observed that the discussion occurring in groups including families and patients and coordinated by a therapist was the most natural and useful format in order to promote changes. The group is the setting where it is possible to see what happens in one's own family thanks to the observation of what happens in the other families, different but similar. This allows a process of substantial modifications of the atmosphere in the single families, who are thus prompted to take the responsibility to actively look for solutions to the experienced problems, without any self-blaming for the patient's illness.

In spite of a large dissemination in Italy and Latin America, and a remarkable theoretical production, the model was not assessed empirically, with the exception of an Italian observational study that found a beneficial effect on family burden, particularly in female caregivers (15).

This model was applied in District 1 of the Department of Mental Health of the Health Trust Roma 1 since 1997, to spread out gradually to the other five Districts in the subsequent years. Since 2011 multifamily groups are held weekly in all the twelve CMHCs of the Department of Mental Health that serves a population of 1,041,220 people. Participation to the groups is systematically registered in Districts 1, 2, and 3 since 2015.

## Methods

This is an observational study based on the registry of those who attended the weekly multifamily group sessions. The registry was organized in such a way that for each meeting all the participants were reported. All group participants were identified according to family they belonged to and all had their sex and date of birth recorded. For patients also the diagnosis was recorded. The results presented here are relative to the data systematically collected from July 2015 to November 2019 in all six CMHCs of Districts 1, 2, and 3.

The six CMHCs where the sessions took place were in the Eastern metropolitan area of Rome (Districts 1, 2, and 3) and were part of the Department of Mental Health of the Health Trust Roma 1. According to administrative data relative to 2017, the total population of the three districts was 560,000 people. District 1 had 1,657 people in charge, corresponding to a treated prevalence of 8.94/1,000 inhabitants, and the corresponding figures were 1,958 people and 11.63 in District 2 and 1,579 and 7.70 in District 3. In District 1 the percentage of users with severe mental illness was estimated 66% and in the other two Districts 52 and 71%, respectively. Districts 1 and 2, in Rome central area, although heterogeneous from a sociodemographic standpoint, are among the most affluent areas of the city. District 3, in the North-East of the city, is a less affluent area. In each District there are two CMHCs, where a multidisciplinary team operates, open 12 h/day from Monday to Saturday. They share an asset of rehabilitation residential facilities, Day Centres and other services.

The psychodynamic multifamily groups are based on an open and free dialogue among participants, and employ three simple rules: 1. each participant speaks one at a time about an issue chosen by him/herself and the others listen to without interrupting; 2. nobody's opinion is considered “right” and all participants are requested to listen to and respect other people's point of view, even when it differs from theirs; 3. participants raise their hand in order to take the floor and take their turn accordingly.

Each meeting has two to three facilitators who ensure the ground rules are followed and help to maintain a climate of openness. They never make any diagnostic evaluation, suggest psychological interpretations, or address issues of possible etiopathogenesis of psychiatric disorders. Sessions are held weekly, and last 90 minutes.

In all CMHCs the multifamily groups are known to all service users and professionals. There are announcements in the reception of the CMHCs, and professionals usually present the possibility to access the groups to the new users. This widespread knowledge is also due to the fact that the groups have been holding since several years. There are no criteria to select patients for the groups. Users are free to join if they want to, independently of diagnosis, severity of the disorder, or age, and even without any specific referral from the treating team. Anyway, the team may suggest a client and their family join the multifamily groups at a certain point e.g., after discharge from an acute ward or residential facility, or in cases with a history of repeated hospital admissions, or when a setback in the therapy occurs. In these cases it is recommended that at least one professional of the team accompanies the participant(s) to the first two-three multifamily group sessions. There is no pre-defined duration and participants can stop going at any time. Generally, 4–6 professionals from the respective CMHCs also attend the meetings.

In each multifamily group there are professionals with different backgrounds and experience. At least one facilitator in each group has specific certified training in multifamily group therapy and receives regular supervision. The delivery of the groups across Rome has become more consistent over time, in part due to a program of international exchange among all the multifamily groups across Argentina, Uruguay, Italy, Spain, and Portugal with Jorge Garcia Badaracco himself, until 2011, when he died.

Duration of participation corresponded to the number of weeks between the first and the last meeting of each participant. The rate of attendance was computed as the percentage of meetings attended in the total number of weeks. Degree of active participation in the meeting was assessed by the facilitators just after the meeting and were based on the frequency of contributions to the discussion.

In the analysis, participants were divided into three groups: family including the patient, only family members without the patient, only patient. These groups were compared as for sex, age and diagnosis of the patient, duration of the participation to the multifamily groups since the first occurrence (divided into quartiles), rate of meeting attendance in the entire period from first occurrence (in quartiles), and degree of active participation shown during the meetings (in quartiles). For the families composed of more than one person, the value used for the duration of participation and the rate of attendance characterizing the family were those of the member who showed the longest duration of participation, and the degree of active participation was the mean of the values reported for each meeting by the same participant.

Associations between the three types of families and diagnosis of the patient, participation and demographic characteristics were tested by means of Pearson's chi-squared test. Analyses were conducted using JMP Pro 15, SAS Institute Inc.

## Results

Between July 2015 and November 2019, a total of 1,044 meetings were held in the six CMHCs, with a mean number of participants ranging between 13 and 31 according to the CMHC. The total number of family units who participated to the multifamily group sessions was 439, corresponding to a total number of 794 persons, and representing about 15% of the severe cases of the three Districts. Family units were represented only by the patient in 180 cases (41%), >1 relative or other close person in 76 cases (17%), and >1 relative/other close person and patient in 183 cases (42%).

The mean number of groups attended by patients was 18.6, by mothers 25.6 and by fathers 21.6. Eighty-six percent of families attended the meetings more than once.

Mean age of participating patients (either alone or with their families) was 42.8 (SD 13.9) and the median was 42 years. 43.6% were female. The most prevalent patients' diagnosis was schizophrenia (169 patients, 38.5%), followed by personality disorders (94, 21.4%) and bipolar and depressive disorders (90, 20.5%).

Mean duration of participation was 68.3 weeks (SD 69.3), and the average rate of presence in the period of participation was 56.5% (SD 34.3). One hundred seventy participants (26.7%) were in the highest quartile of degree of active participation, and 90 (21.1%) in the lowest quartile. Brothers or sisters showed the highest degree of active participation, followed by mothers, patients and fathers.

The three groups (Table 1) differed according to sex and age. Patients participating alone tended to be older than those of families participating with or without the patients themselves. Family units composition was also associated to patient's diagnosis. The presence of a diagnosis of schizophrenia and, to a lesser extent, personality disorder in the index patient was associated to participation of families including the patient. Families including the patient also showed the longest duration of participation, whereas there were no statistically significant differences in rate of attendance and degree of active participation during the meetings according to family unit composition.

**Table 1:** Composition of 439 families according to characteristics of the patient and participation of the family.

	**Family composition**						
	**Patient only (180)**		**Family member(s) only (76)**		**Patient and ≥ 1 family member(s) or other (183)**		
	**No**.	**%**	**No**.	**%**	**No**.	**%**	**Chi-square**
							***p***
**Sex**							
Female	83	46.1	30	39.5	78	42.3	13.347 <0.01
Male	97	53.9	41	54.0	101	55.5	
**Age**							
<30	16	8.9	18	25.7	45	24.9	76.975 <0.001
30–39	22	12.3	23	32.9	61	33.7	
40–49	59	33.0	9	12.9	50	27.6	
50–59	49	27.4	8	11.4	19	10.5	
≥60	33	18.4	12	17.1	6	3.3	
**Diagnosis**							
Schizophrenia spectrum and other psychotic disorders	53	29.4	32	42.1	84	45.9	36.109 <0.0001
Bipolar and depressive disorders	51	28.3	10	13.2	29	15.9	
Personality disorders	31	17.2	25	32.9	38	20.8	
Anxiety and somatic symptoms disorders	34	18.9	6	7.9	14	7.7	
Others	11	6.1	3	3.9	18	9.8	
**Duration (weeks since first meeting)**							
>8	52	29.1	29	38.7	40	22.0	8.033 <0.05
8–40	51	28.5	16	21.3	32	17.6	
41–122	41	22.9	16	21.3	50	27.5	
≥123	35	19.6	14	18.7	60	33.0	
**Rate of participation**							
<30%	44	25.0	22	29.3	40	22.1	10.400 ns
30–51%	48	27.3	17	22.7	41	22.7	
52–88%	39	22.2	13	17.3	60	33.2	
>88%	45	25.6	23	30.7	40	22.1	
**Degree of active participation**							
≤ 1.3 (low)	51	29.0	23	32.4	40	22.2	6.489 ns
1.4–1.9	70	39.8	27	38.0	77	42.8	
2.0–2.2	18	10.2	11	15.5	20	11.1	
2.3–3 (high)	37	21.0	10	14.1	43	23.9	

Participation was longer than 122 weeks in 52 family units (31%) where the patient had a diagnosis of schizophrenia, 27 (29%) families where the patient had a diagnosis personality disorders, and 9 (32%) families where the patient had a diagnosis of other disorders. Diagnosis was not associated to rate of attendance. As expected, rate of attendance tended to be higher when the duration of participation was shorter, with very few people being able to maintain a very high rate when their attendance lasted more than 40 weeks. However, 38.5% of families with the longest duration showed a rate between 55 and 88% (Table 2).

**Table 2:** Relationship between duration of and intensity of participation in 375 families with more than one week of participation.

	**Duration (weeks)**				
**Rate of participation**	**<8**	**8–40**	**41–122**	**≥123**	**Chi-square** ***p***
<30%	3 (5.0%)	26 (26.3%)	44 (41.1%)	32 (29.4%)	89.559
30–51%	16 (26.7%)	30 (30.3%)	26 (24.3%)	34 (31.2%)	<0.0001
52–88%	11 (18.3%)	29 (29.3%)	30 (28.0%)	41 (38.5%)	
>88%	30 (50.0%)	14 (14.1%)	7 (6.5%)	1 (0.9%)	

## Discussion

Psychodynamic multifamily groups started in 1997 in several CMHCs in Rome, to be implemented regularly on a weekly basis starting from 2011. Since then, a considerable number of family units and individuals regularly attended multifamily groups every week in each CMHC. We have found a good rate of attendance persisting across the years of observation, with new entries and a portion of long-term participants. The majority of participants had experience of severe and persisting disorders, with the diagnoses of schizophrenia and personality disorders largely represented. These diagnoses were also associated to long duration of participation.

Although the multifamily groups were widely known and easily accessible to all with no waiting list, the families attending the groups corresponded to only the 15% of the severe cases. It is possible that the demand for such interventions exceeds the response from the services, but it is also likely that other factors, not related to the limited offer, can explain the small proportion of families involved, like patients and family members not willing to participate due to wish of privacy, limited trust in the mental health services, fear of a too high emotional demand, or, more simply, for reasons like lack of time and too long distance from home (16, 17).

Patients participating alone were as many as family units including the patient. Family units consisting of family members or close persons without the patient were much fewer and showed shorter duration and lower degree of active participation during the meeting, suggesting that the groups may work better when the patient is there, in agreement with studies assessing the effectiveness of psychoeducation (18). The high number of patients who attended alone suggests that such participation represented a free personal choice and corresponds to a self-perceived need. Anyway, the multifamily groups represent the only setting where patients can meet with other people and professionals in a free still structured way, and with not strictly therapeutic objectives.

Whereas, patients were the most represented among participants, mothers participated more frequently than fathers, and fathers seldom showed active participation to the groups, thus confirming a different attitude in mothers and fathers. This more active participation in mothers may be linked to the effect observed in groups of families of children with a first episode of psychosis, and concerning the quality of participation and coping strategies elicited by mothers and fathers, where, in the framework of overall levels of psychological distress and similar beliefs about the illness, mothers showed more emotion-focused coping strategies, like sharing how they feel (19).

Heterogeneity in rate of attendance, duration, type of participants and composition of the families attending the multifamily groups can be related to the open nature of the setting, where there was no selection or referral procedure and all were free to attend. Moreover, the high participation over the years may reflect that the groups are fulfilling a need for long lasting support for some families and patients that may not be available elsewhere in the mental health system. We also found a remarkable portion of families with long duration of participation who could maintain a reasonable rate of attendance, suggesting that even in the long run families attended rather regularly, likely continuing to perceive a benefit. It was shown that longer duration in itself, more than the actual number of sessions, ensured more improvement in patients (20). This is reassuring, since in this sample long participation was shown by a significant numbers of family units with lower rates of attendance.

The implementation of family interventions in the treatment of severe psychiatric disorders, although considered effective major components of care, is still extremely limited (21). This might be due to severe workload, pressure on specialized services, organization pitfalls, limited staff's training and skills, as well as to a pessimistic view of recovery for people with severe mental illness (22). We have described an experience of systematic implementation of multifamily groups in a metropolitan area with more than one million inhabitants. This was possible thanks to several factors. Specific indications from the Direction of the Department were coupled with wide interest and compliance from services and staff, sustained by a sort of spontaneous cultural osmosis, according to a top-down bottom-up integration. This is suggested by the large participation to the groups of professionals not involved in conduction and facilitation of the groups, that occurred in spite of the increasing pressure on mental health services and the dramatic deprivation of resources. According to data collected on a national basis, service staff of the Region where this experience was conducted was reduced by 68% in the period 2015–2018 (23). This is consistent with the idea that implementation of family-based services is affected not only by structural and organizational factors (5) but also by factors connected to a cultural shift shared by leaders and first line professionals (24). Such process may represent a reframing of the therapeutic alliance in two ways: on one side, by reducing the influence of the paradigm based on biological models of mental disorders and focusing on the social ground where patients and families live; on the other side, by overcoming the blaming attitude toward the dysfunctional aspects in the families, which contributes to “a loss of trust in services and strained relationships between professionals and families” (21, p. 9).

The permanent availability of the multifamily groups also challenges the gap between research and practice. The assessment of long-term effectiveness of family interventions is based mainly on the results of 1–2-year follow-ups, and the issue of whether it is sustained after treatment termination is mixed (24–26). Such a crucial issue likely pertains to all psychosocial interventions, usually offered on a time-limited basis to individuals with persistent long-lasting problems and needs. It has been suggested that, at least in the most complex cases, continuity of such treatment should be assured through ongoing support, even informal (27), or through an open-ended multifamily group structure for families in need (28).

One more issue is related to the need to combine flexibility and continuity in the delivery of the services in order to develop truly community-focused recovery-oriented interventions, dealing with the “real world” of patients and their families and providing treatment that is flexible and tailored to the individual needs (29–31). In this perspective, Glynn et al. (24) envisaged a possible shift in involving families as an influence of the recovery approach, from a “behavioral family management,” with the emphasis on negative outcomes rather than building on strengths, to a consumer-driven support approach, with attention to increasing communication and cooperation between mental health professionals and families. The characteristics of continuity and flexibility of the multifamily groups of this study are consistent with a paradigm of dialogue among professionals, consumers and families in a recovery perspective. Consistently with this, the role of the facilitator is closer to that employed in the Open Dialogue approach (32) rather than in psychoeducation. In fact, notwithstanding the multifamily groups share several core strategies with psychoeducation as summarized by Pharaoh et al. (6), like building alliance, reduction of adverse family atmosphere and feelings of guilt, attainment of desirable change in relatives' belief systems, they also show marked differences. Namely, there is no focus on information/education and problem solving, and drug compliance and clinical stabilization are not directly pursued. Rather, change is promoted more through active participation, highlighting and acceptance of the multiple points of view, enhancement of self-righting and self-determination. Consequently, facilitators did not play the role of experts who educate and answer to questions, rather they favored an exchange of views involving as many people as possible, where everybody's standpoint is taken into serious consideration. Moreover, listening and paying attention to different ideas coming directly from consumers and family members in an unfiltered way allow facilitators and professionals to learn about how services could best answer to people's needs as directly perceived and expressed by them.

This study describes an activity as it is conducted in the routine. To our knowledge, few experiences of multifamily groups regularly and persistently available in mental health community services were previously described. Anyway, this report is plagued by several limits. First of all, it is a straightforward account of the implementation of the multifamily groups based on a limited set of variables with no information of outcome indicators, therefore preventing an analysis of effectiveness on patients' and families' mental health and quality of life. Duration is only a proxy of the real one, since data presented only cover the period from 2015 to 2019 and are therefore not comprehensive.

In spite of these limits, our results demonstrate that it is feasible to provide and facilitate well-attended multifamily groups over the long term in an inner city area. More research is needed to establish their effectiveness in terms of clinical and social outcomes.

## Data Availability Statement

The raw data supporting the conclusions of this article will be made available by the authors, without undue reservation.

## Ethics Statement

The study protocol was approved by the Ethics Committee of Lazio 1 (Comitato Etico Lazio 1, https://www.comitatoeticolazio1.it/, Prot. N.1441, Date 27/11/2020). Informed voluntary written consent was obtained from every individual participants who were screened for study eligibility.

## Author Contributions

AM, AN, and FR: study design. AM and FR: study coordination and implementation. BD'A, BG, and RE: data collection and analysis. AM and BD'A: drafting the manuscript. All authors contributed to the article and approved the submitted version.

## Conflict of Interest

The authors declare that the research was conducted in the absence of any commercial or financial relationships that could be construed as a potential conflict of interest.
